# The Feasibility and Acceptability of an mHealth Conversational Agent Designed to Support HIV Self-testing in South Africa: Cross-sectional Study

**DOI:** 10.2196/39816

**Published:** 2022-12-12

**Authors:** Xolani Ntinga, Franco Musiello, Alfred Kipyegon Keter, Ruanne Barnabas, Alastair van Heerden

**Affiliations:** 1 Centre for Community Based Research Human Sciences Research Council Pietermaritzburg South Africa; 2 Department of Clinical Sciences Institute of Tropical Medicine Antwerp Antwerp Belgium; 3 Department of Applied Mathematics, Computer Science and Statistics Ghent University Ghent Belgium; 4 Department of Medicine Harvard Medical School Boston, MA United States; 5 Division of Infectious Diseases Massachusetts General Hospital Boston, MA United States; 6 South African Medical Research Council/WITS Developmental Pathways for Health Research Unit Department of Paediatrics, School of Clinical Medicine, Faculty of Health Sciences University of the Witwatersrand Johannesburg South Africa

**Keywords:** HIV, HIV self-testing, HIVST, chatbot, conversational agents, mobile health, mHealth, mobile phone

## Abstract

**Background:**

HIV testing rates in sub-Saharan Africa remain below the targeted threshold, and primary care facilities struggle to provide adequate services. Innovative approaches that leverage digital technologies could improve HIV testing and access to treatment.

**Objective:**

This study aimed to examine the feasibility and acceptability of *Nolwazi_bot*. It is an isiZulu-speaking conversational agent designed to support HIV self-testing (HIVST) in KwaZulu-Natal, South Africa.

**Methods:**

Nolwazi_bot was designed with 4 different personalities that users could choose when selecting a counselor for their HIVST session. We recruited a convenience sample of 120 consenting adults and invited them to undertake an HIV self-test facilitated by the Nolwazi_bot. After testing, participants completed an interviewer-led posttest structured survey to assess their experience with the chatbot-supported HIVST.

**Results:**

Participants (N=120) ranged in age from 18 to 47 years, with half of them being men (61/120, 50.8%). Of the 120 participants, 111 (92.5%) had tested with a human counselor more than once. Of the 120 participants, 45 (37.5%) chose to be counseled by the female Nolwazi_bot personality aged between 18 and 25 years. Approximately one-fifth (21/120, 17.5%) of the participants who underwent an HIV self-test guided by the chatbot tested positive. Most participants (95/120, 79.2%) indicated that their HIV testing experience with a chatbot was much better than that with a human counselor. Many participants (93/120, 77.5%) reported that they felt as if they were talking to a real person, stating that the response tone and word choice of Nolwazi_bot reminded them of how they speak in daily conversations.

**Conclusions:**

The study provides insights into the potential of digital technology interventions to support HIVST in low-income and middle-income countries. Although we wait to see the full benefits of mobile health, technological interventions including conversational agents or chatbots provide us with an excellent opportunity to improve HIVST by addressing the barriers associated with clinic-based HIV testing.

## Introduction

### Background

Identifying patients with undiagnosed HIV and preventing new HIV infections remain critical public health issues. To reduce HIV incidence, global strategies emphasize early diagnosis, immediate treatment, and ongoing viral suppression for those living with HIV [1]. Despite the expansion of HIV testing services (HTSs) in sub-Saharan Africa, one-fifth of those aged between 15 and 64 years remain undiagnosed. Men, adolescents aged between 15 and 19 years, and adults aged ≥40 years continue to be infected with HIV owing to HIV testing gaps, which contribute to poor health outcomes and continued HIV transmission [2,3].

South Africa’s health system is characterized by a quadruple burden of communicable, noncommunicable, maternal and child health, and injury-related disorders [4-7]. Primary health care (PHC) facilities in South Africa grapple with screening, initiating, and treating people with HIV, coupled with high incidence of tuberculosis, high maternal and child mortality levels, and growing burden of noncommunicable diseases [4,6]. As a result of the COVID-19 pandemic, facility-based HTS face new barriers in South Africa and elsewhere [8,9]. Currently, several approaches are promising in relieving some of the strain PHC facilities face, including HIV self-testing (HIVST), Chronic Medicines Dispensing and Distribution, community-based adherence clubs, and quick pharmacy pickups [7,10-12].

Despite these critically important and valuable initiatives, we suggest that, rather than incremental improvements within the existing framework of primary care, what is required is a reimagination of primary care that places digital services at the entry point of the health system instead of relying exclusively on human resources. Innovative approaches that leverage digital technologies could benefit populations that are not currently served by existing approaches. Although many of the benefits of mobile health (mHealth) have not yet materialized as hoped, the ubiquity of mobile phones, increasing availability of point-of-care health devices and screening tests, ability to collect and availability of large amounts of data about human behavior, and advances in machine intelligence make this proposed approach a possibility in the near future. HIV self-screening is an excellent test case for nonhuman intervention in the PHC system. Several issues with the current model of care deter people from getting tested [9]. Routine HTS in South Africa primarily uses a provider-based approach [13]. In this approach, individuals must visit an HIV testing location, such as a hospital or community center [9].

Although there have been gains in increasing access to HTS, barriers to the uptake of facility-based HTS include stigmatizing norms, discrimination from health care workers, distance to health facilities, and direct and indirect service use costs [9,14-17]. Innovative strategies to overcome these barriers will be critical to achieving the Joint United Nations Programme on HIV/AIDS *95–95–95* goals.

HIVST is a relatively new approach that provides an opportunity to reach, test, and diagnose or prevent infection among populations previously considered to be unreachable, even during the COVID-19 pandemic, owing to people’s ability to self-test at home [15]. The World Health Organization recommends HIVST as an additional approach to provide HTS to help close this testing gap by increasing access and acceptability for HIV testing [13]. HIVST presents a private, convenient, and confidential approach to providing HTS that removes some of the barriers to routine HTS by allowing people to collect their samples and receive their results in the privacy of their homes, without interacting with a health care professional [9]. HIVST also reduces costs and saves time for the health delivery system and end user by triaging out the patients who are HIV-negative [18-20]. To support the use of HIVST in South Africa, guidelines for HIVST implementation were issued by the National Department of Health in February 2018 [9]. However, there are several concerns related to HIVST, such as lack of a formal pipeline for users to self-report their results or be linked to care following the self-test, potential of mental health risks associated with testing positive without counseling support, potential inability of testers to cope with their result, and that patients who undergo HIVST are less likely to access care [21,22]. Strong mobile phone penetration in low-income and middle-income countries has led to the development of various mHealth interventions to complement HIVST [23,24]. These include telephone hotlines, SMS text messaging interventions, internet-based platforms, and mobile apps. South Africa’s mobile phone penetration and access to the internet is strong, with 89 (40%) of the households nationally having a mobile phone. In KwaZulu-Natal, 87.5% of households own mobile phones. Moreover, the national proportion of households with internet access was 74.1% in 2020. In the same year, 72.3% of households in KwaZulu-Natal had access to the internet [25].

Exploration of conversational agents in a health care setting suggests that users accept [21,22] and can form a working alliance with [21] embodied conversational agents. Examples of such agents include Florence, which was developed in the United Kingdom by the National Health Services as a digital solution for patient self-management and adherence through user-friendly, intelligent messaging that improves health outcomes—freeing up time and resources for clinicians and the health care system [26]. In addition, there is Molly, an empathy-based conversational platform developed by Sensely Corporation for linking people to care and managing chronic conditions, which is currently available in Japan, the Philippines, the United Kingdom, and the United States [27]. In addition, KOKO (developed by KoKo Incorporated) is a platform used to manage mental health, which is currently available in the United States [28]. In South Africa, mHealth apps developed to support HIVST and reporting include the AspectTM HIVST app designed by SystemOne LLC [22] and the Ithaka mobile app developed by Aviro Health [24]. These conversational agents use a natural language understanding (NLU) engine to understand and respond to human interaction. NLU makes it possible to identify underlying user intents and enables the extraction of context, meanings, and domain-specific entities in users’ utterances. NLU typically identifies three aspects in a sentence: (1) intent, which is done by mapping users’ utterances to a specific class that allows digital web-based assistants to decide a response or action; (2) entities that illustrate important information such as date, times, and locations; and (3) contexts, which correspond to the context of the object the user is referring to [29]. A chatbot’s accurate response to users’ input requires combining these intents, entities, and contexts. Although there is increasing interest in the use of NLU-driven conversational agents in the health care context, the extent to which people find them acceptable for different uses needs further evaluation.

### Objective

In this study, we examined the feasibility and acceptability of *Nolwazi_bot*, an isiZulu-speaking conversational agent designed to support HIVST in South Africa. The work on Nolwazi_bot began with the pilot study in 2017 [30], followed by a grant application that was successful in 2020. Upon commencement of data collection, there was already a trend in the development of digital interventions to support HIV, which is continuing to grow [31-35] owing to their agility and scalability because of low implementation, long-term recurring costs, and opportunity to reduce stigma and confidentiality concerns even among hard-to-reach populations [36]. Some of the recent studies on digital HIV interventions in South Africa [22,24] have been conducted in inner-city Johannesburg, which is South Africa’s largest city. This study was conducted using a community-based approach in the rural Vulindlela subdistrict in KwaZulu-Natal province. In South Africa, KwaZulu-Natal is the province with the highest HIV prevalence, with evidence that uMgungundlovu is one of the districts with the highest prevalence (30%) in the country [37]. Given the different population of this chatbot feasibility study compared with the others conducted in South Africa, this study is the first to test a chatbot in a rural setting that has high HIV prevalence in South Africa. This study is also aiming to add to the literature that supports the idea that digital innovations are highly acceptable across diverse settings.

## Methods

### Study Design

This cross-sectional pilot study was conducted from December 2020 to April 2021. A convenience sample of 120 consenting adults were recruited from the Vulindlela subdistrict (uMgungundlovu district) in KwaZulu-Natal, South Africa. Recruitment was undertaken by a trained community outreach team who spoke to the public about this study. Those interested were screened against inclusion and exclusion criteria and then brought to the Human Sciences Research Council’s Sweetwaters office to provide consent and complete the study. Participants were included if they had previously tested for HIV with a human counselor at any time in their life, were aged ≥18 years, resided in Vulindlela or a neighboring community, could use a smartphone to chat with the Nolwazi_bot (chatbot), and were able to provide written informed consent. Participants were excluded if they did not meet the inclusion criteria, had any condition that may have interfered with the testing process (such as intoxication or poor vision), or reported being HIV-positive. App feasibility is often assessed in a variety of ways. This study assessed feasibility by considering the following three criteria: (1) participants’ acceptance of using the app (the chatbot in this study), (2) the participant’s ability to complete the task on the app, and (3) the ability of the app to perform the required task [38]. For this study, these criteria were operationalized as follows: (1) the participants’ willingness to undergo an HIVST guided by a chatbot, (2) the participants’ ability to interact with the chatbot and follow the instructions of testing, and (3) the chatbot’s ability to guide the participants to conduct HIVST and interpret their results. We also explored the socioeconomic status using the assets (electric stove, tap water, and car) available at participants’ homes. We sorted individuals by the asset index and established cutoff values for percentiles of the population. Then, we assign households to a group based on their value on the index. For expository convenience, we refer to the bottom 40% as *poor*, the next 40% as *middle*, and the top 20% as *rich* [39].

### Ethics Approval

Ethics approval was obtained from the Human Sciences Research Council Research Ethics Committee (reference number 13/22/11/17). Trained study staff obtained written informed consent from all study participants using an information sheet and informed consent document approved by the Human Sciences Research Council Research Ethics Committee. The informed consent form was available in English and isiZulu. Participants were given reimbursement of R150 (equivalent to US $8.68) for their participation in the study.

### Agent Development

**Figure 1:**
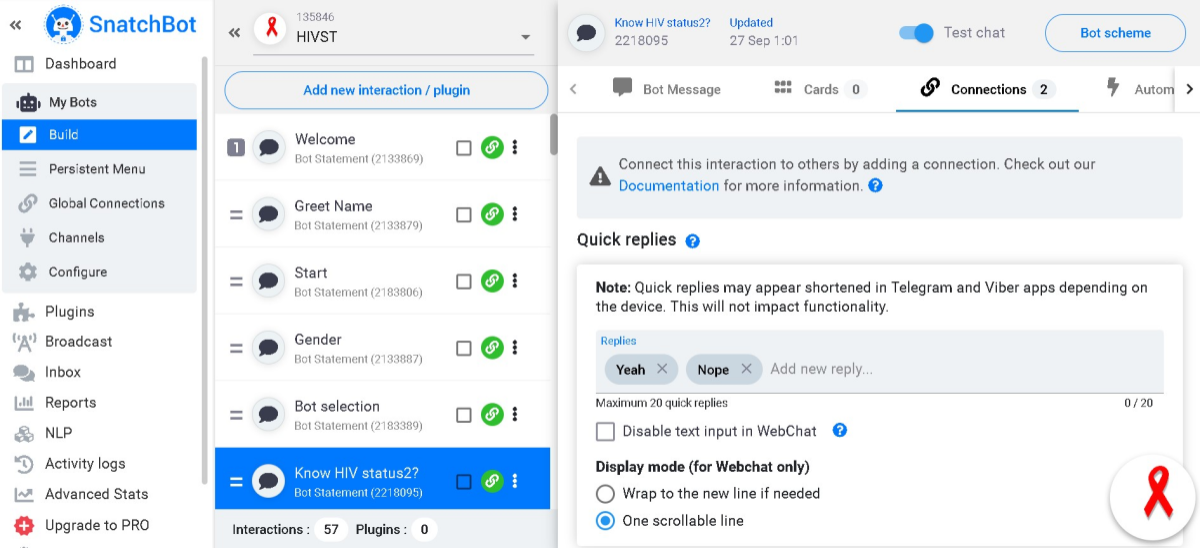
The building scheme of Nolwazi_bot.

The authors adapted a previous chatbot they had created using dialogue flow. Several commercially available websites that offered Natural Language Processing as a service were reviewed for suitability. Criteria used in the evaluation of the offerings were (1) integration with chat clients popular in South Africa (such as WhatsApp, WeChat, and Telegram), (2) graphical user interface for chatbot design and training, (3) ability to export model for publication, (4) protection of personal information regulatory compliance, and (5) offering webhook integration to interface with other services and allow the addition of anticipated future functionality. SnatchBot was selected, and Nolwazi_bot was designed and built on SnatchBot according to the Center for Disease Control’s guidelines for the provision of HIV counseling and testing in a nonclinical setting [40]. The content of the chatbot was reviewed by a nurse working at the Human Sciences Research Council in English and isiZulu to assess its compliance with the self-testing guidelines described in the South African National HIV Testing Services Policy [41]. The content was also reviewed by bilingual translators at the Human Sciences Research Council to ensure that the language was culturally appropriate to use and easy to understand. The development of Nolwazi_bot followed the principles of the SnatchBot platform (drag and drop and code-free design). SnatchBot provides an in-built editor that can be used to develop a simple or complex conversation with action buttons and translations. In addition, SnatchBot allows designers to create many interactions in relation with activities of the chatbot. During development, we created interactions that describe the predefined response patterns from the chatbot (including messages, videos, graphs, etc) after a user has said something. For example, in building an interaction between the chatbot and the user to introduce themselves, we created an interaction called *welcome*; if a person greets the chatbot it will reply with predefined response, that is, *welcome*. In a similar way, many other interactions were created, including name, bot selection (selecting a counselor), goodbye, known HIV status, HIV test results, and linkage to care, among others. Using SnatchBot, we created a design consisting of interactions and subjects. Figure 1 shows some part of the building scheme of Nolwazi_bot.

Nolwazi_bot was designed to have 4 personalities that users could choose to be their counselor during the session. Of the 4 personalities, 2 were aged between 35 and 50 years (a middle-aged man and a middle-aged woman), and the other 2 personalities were aged between 18 and 25 years (a man and a woman in their youth). During the session, the older personalities spoke more formally in isiZulu, whereas the younger ones used a mixture of English and isiZulu.

### Testing Procedure

#### Overview

The trained researcher obtained voluntary written informed consent from the participants in a private room. Each participant was temporarily provided with a Samsung J4 mobile phone running Android 8.1.0, with the Telegram messaging app preinstalled on the phone and an accompanying HIVST kit. The sealed test kit contained an English brochure with instructions for use as part of the standard packaging; however, the participant was requested to perform HIVST by following the isiZulu instructions on Nolwazi_bot and only use the instructions on the HIVST kit if requested by the chatbot. Once the participant was alone in the room, they opened the Telegram messaging app, searched for Nolwazi_bot, and opened the chatbot. Then, it greeted them and told them that they could choose to speak to a human at any point during the conversation by typing *help*, *I need help*, or *please help*. Then, the chatbot introduced 4 people, one of whom they could choose as their counselor for the session. Overall, 4 images were presented by the chatbot, which included 2 young individuals (aged 18-25 years; 1 man and 1 woman) and 2 older individuals (aged 35-50 years; 1 man and 1 woman). Stock photos were used to represent the personalities. For the young personalities, the language used by the bot was more colloquial and a mixture of isiZulu and English to represent how the young demographic group speaks in the study community, and it is typical of what would be used by young HIV counselors. For the older personalities, the chatbot converses in professional isiZulu, which did not include any English words.

**Figure 2:**
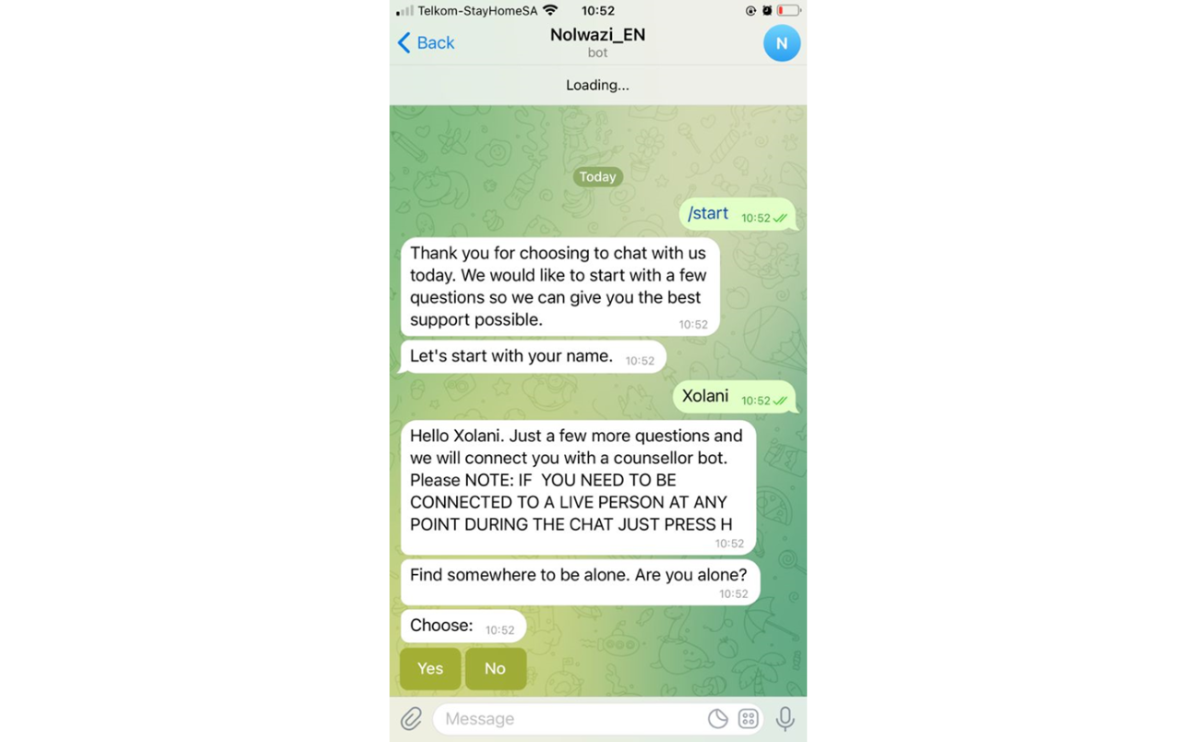
Interaction between the tester and Nolwazi_bot in the Telegram messaging app.

The chatbot guided them through the conversations and emphasized that they could choose not to test if they did not feel comfortable or were not ready to test. Once they were ready to test, the chatbot provided them with a link to a video about using the kit and interpreting the result. The BioSure HIVST kit (BioSure Ltd) was used for the study, as it is already available and used in South Africa. Obtaining the sample takes 2 to 3 minutes, followed by a 15-minute waiting period for results to be produced. Then, participants would interpret their results alone and provide the app with their test results. A status-neutral approach was used for testing, with participants receiving a negative test result being asked if they wanted to learn more about pre-exposure prophylaxis (PrEP) options in the area. Participants who are HIV-positive were asked if they needed assistance with disclosing the result to family, friends, or their partner or if they required assistance in linking to care. Participants were informed that they still needed to visit the clinic to get tested again and confirm their positive result, according to South African HIVST guidelines. Participants were invited by Nolwazi_bot to take a picture of their test kit that confirmed their HIV-positive results and present it to the clinic when they link to care. Figure 2 shows an example of the interaction between the tester and Nolwazi_bot in the Telegram messaging app. Following the test, the researcher asked the participants to participate in a posttest survey.

#### Posttest Survey

A face-to-face, interviewer-led, posttest structured survey (Multimedia Appendix 1) was conducted to obtain user feedback on their experience with Nolwazi_bot. The posttest survey was paper-based, and the pragmatic reason for having a paper-based survey was that the researchers would have had to create a separate survey bot, as the current bot was created for HIV testing. The investigators had planned to use the system usability scale by Brooke [42], which is a standardized scale for system or product usability assessment. However, after reviewing the scale, it was decided that considerable adaptation of the items would be required for this study. Therefore, we decided to use an adapted, unvalidated scale to assess usability. Unfortunately, resources were not available to perform cognitive interviews or other validation procedures before its use.

The posttest survey questions in this study were designed to support the understanding of the user experience of HIVST guided by a chatbot compared with a human counselor. Several questions were dichotomous *yes* and *no* questions, one was a Likert scale question, one was a scale question, and some were asking participants to choose between 2 different options. Participants were also asked open-ended questions regarding their perception of how human the conversation felt, why they preferred having a conversation with a particular sex, and the advantages and disadvantages of the chatbot. Open-ended questions were included in the study owing to the exploratory nature of the study. All participants (120/120, 100%) were invited to participate in the poststudy survey. Participants were eligible to participate if they provided consent and had completed the HIVST using the chatbot. All participants (120/120, 100%) participated in the poststudy survey. After the test and posttest survey were completed, the participants returned the phone to the researcher, and the used HIVST kits were disposed according to guidelines.

### Data Analysis

All data extracted from the survey questionnaire (paper-based) and downloaded from the counseling chatbot were entered into the SPSS (version 25.0; IBM Corp) software. Exploratory descriptive statistics including frequencies and proportions were generated for demographic information, questions about HIV testing, chosen counselor, and HIV status, and PrEP information was described using frequencies and percentages. The open-ended questions were coded into ATLAS.ti and analyzed thematically. The qualitative data provided categories that supported the quantitative responses and allowed for better understanding of the participants’ quantitative responses. For example, there was a question that asked participants “did it feel like a real person was replying?” We produced counts of the number of people who reported that the chatbot was similar to a real person; then, a follow-up qualitative question asked why it felt similar to a real person or why it did not feel similar to a real person. These qualitative responses were then used to provide themes to support the *realness* of the chatbot. A chi-square test was used to assess associations between categorical variables.

## Results

### Overview

Between December 2, 2020, and April 9, 2021, we screened 126 participants for the study, of whom 4 (3.2%) were not eligible owing to reported known HIV-positive status and 2 (1.6%) were not able to use a smartphone. A final sample of 95.2% (120/126) of participants aged 18 to 47 years, with median age of 24 (IQR 21.8-28) years, performed HIV self-test using the Nolwazi_bot. The sample was approximately equally divided across sexes. Overall, two-thirds of the participants (81/120, 67.5%) had secondary or high school education, and 92.5% (111/120) had tested more than once with a human counselor before testing with the chatbot. Up to 37.5% (45/120) of the participants chose a woman aged between 18 and 25 years to have a counseling session with. Participants conducted HIVST guided by the chatbot, and 17.5% (21/120) of them tested positive. These participants were provided with a referral to visit their preferred nearest clinic for a confirmatory test and linked to care. Thereafter, the community outreach team at the Human Sciences Research Council conducted follow-ups to check their linkage to care. Participants who tested negative were offered the option to learn more about PrEP, and 82.8% (82/99) of them wanted to know more about PrEP. Many participants (49/120, 40.8%) were from middle socioeconomic background, followed by poor socioeconomic background (48/120, 40%). There were no differences in responses by participants across variables when comparing HIV status. Further demographic data are presented in Table 1.

**Table 1:** Demographics characteristics of participants (N=120).

Characteristics		Value, n (%)
**Sex**		
	Male	61 (50.8)
	Female	59 (49.2)
**Education**		
	Primary school	2 (1.7)
	Secondary or high school	81 (67.5)
	Tertiary institution	33 (27.5)
	None	4 (3.3)
**Times tested for HIV**		
	1	9 (7.5)
	2-5	47 (39.2)
	5-10	28 (23.3)
	>10	36 (30)
**Chosen counselor (age [years]; sex)**		
	18-25; male	26 (21.7)
	18-25; female	45 (37.5)
	35-50; male	24 (20)
	35-50; female	25 (20.8)
**HIV test outcome**		
	Negative	99 (82.5)
	Positive	21 (17.5)
**SES^a^**		
	Poor (lower 40%)	48 (40)
	Middle (middle 40%)	49 (40.8)
	Rich (upper 20%)	23 (19.2)
**Response to question about whether they would like to know about PrEP^b^ (n=99)**		
	Yes	82 (83)
	No	17 (17)

^a^SES: socioeconomic status.

^b^PrEP: pre-exposure prophylaxis.

### Chatbot Experience

After completing HIVST guided by the chatbot, participants were asked to assess the experience of undergoing HIV testing with the assistance of a chatbot compared with that of a human counselor. Of the 120 participants, most participants (n=95, 79.2%) indicated that their HIV testing experience with a chatbot was much better than that with a human counselor, 14 (11.7%) felt that the experience was approximately the same, and 7 (5.8%) felt that the experience was slightly better. Overall, 1.7% (2/120) of the participants felt that the experience was much worse than that with a human counselor.

### Realness of Chatbot Conversations

Participants were asked whether they felt the counseling support they received during their testing to be similar to that obtained while talking to a real person. Of the 120 participants, 93 (77.5%) participants reported that they felt as if they were talking to a real person because the responses were in a tone that they would normally experience when talking to a person and the choice of words was similar to what they use in daily conversations. Other participants felt that the answers were correct and followed the order of pretest and posttest counseling that they usually participate in at clinics and hospitals. Of the 120 participants, 15 (12.5%) participants said that the counseling session did not feel as if they were chatting with a real person, citing that the chatbot replies were quicker than how people would respond, they did not have an opportunity to ask other questions and had to stick to the conversation, and they had to read all the chatbot responses with no option to use voice to record their own responses. Overall, 10% (12/120) of the participants did not respond to the question.

### Advantages of Chatbot-Supported HIVST Compared With Testing With a Human Counselor

Participants were asked to provide the advantages of chatbot-supported HIVST compared with testing with a human counselor, if they felt they were any. Participants provided many responses; the responses were grouped into 5 broad categories that captured the responses. Of the 120 participants, 28 (23.3%) said that the chatbot provided them with a safe space. Participants mentioned that they do not feel vulnerable and exposed, which makes it easy to communicate with confidence and honesty, without fearing the counselor. The chatbot allows testers to answer questions in comfort without having to think about the other person (as there is no other person), and it allows testers to carefully answer in their own time, without having to worry about wasting a (human) counselor’s time. Participants also mentioned that the bot does not criticize or judge them based on their sexual activity, which makes them feel safe, and they have time to adjust to the counseling session without the nurse being in a hurry to see the next client.

Of the 120 participants, 27 (22.5%) participants reported that an advantage of the chatbot is that it offered HIV testing and counseling (HTC) that is confidential, because there would not be any unintended disclosure as only they will know their HIV test outcome, whereas at the clinic, it is possible for a nurse to talk to someone about a person’s status. Of the 120 participants, 15 (12.5%) participants said that the advantage of the chatbot was its functionality; they mentioned that the chatbot asked the right questions and the counseling was conducted in an empathetic and polite manner and educated them about things they did not know, such as acute HIV infection, PrEP, and information about viral suppression for individuals who are HIV-positive. Of the 120 participants, 12 (10%) participants said that the chatbot was efficient. Participants mentioned that it is fast and saves a lot of time, given that it can work at a fast pace if desired, unlike a human counselor, who will decide the pace of the HTC. In addition, participants indicated that they do not have to spend the (usual) entire day at a clinic to know their results; instead, in 30 minutes, they can know their status and take the next steps. Of the 120 participants, 9 (7.5%) participants felt that the chatbot was easy to use, and they indicated the following:

It is very easy to use, less stressful very understandable, it is the best and very advanced product one could ever wish for.

Participants also indicated that chatbot-assisted HIVST is a good approach to HTC owing to the prevalent high use of cell phones and, in particular, social media. Of note, 24.2% (29/120) of the participants did not have any advantages to provide when asked about their chatbot experience.

### Disadvantages of Chatbot-Supported HIVST Compared With Testing With a Human Counselor

Participants were also asked to provide the disadvantages they noticed when using the chatbot in comparison with performing HTC with a human. Of the 120 participants, 11 (9.2%) participants said that the chatbot lacked empathy in comparison with a human counselor. Furthermore, participants said that if they test HIV-positive, they could kill themselves because they will not receive the same comfort as provided by a real counselor. Moreover, participants indicated that if they exhibit suicidal ideations, the chatbot will not be able to intervene, and they would, in this instance, need to talk to a human counselor as the chatbot would not be able to provide verbal comfort, show feelings, or provide physical comfort such as a hug. Another disadvantage mentioned by 5% (6/120) of the participants was that the conversation with the chatbot was unidirectional. These participants indicated that they felt they could not ask questions during the counseling session. It is worth mentioning that none of the participants (0/120, 0%) elected to speak to a human counselor despite being informed that they could do so at any point during their session. Another point made by participants regarding the unidirectional conversation was that the chatbot will not change its response, even if the response is deemed to be unsatisfactory. Of the 120 participants, 4 (3.3%) participants reported the disadvantage of it being easy to make a mistake when chatting with a chatbot, as some may not be able to follow the instructions correctly and, consequently, make a mistake with the interpretation of their HIV results. Interestingly, 82.5% (99/120) of the participants did not have any disadvantages to provide when asked about their chatbot experience.

### Counselor Preference

Chatbot-supported HTC compared with human HTC was evaluated on a scale of 1 to 10, with *1* being terrible and *10* being brilliant. Of the 120 participants, 12 (10%) participants did not respond to this question. Among those who responded, the average score was 9.32 (SD 1), with minimum score of 6 and maximum score of 10. Preference for the counselor among the participants was assessed. The participants were asked whether they would prefer a male or female conversational agent and the reason for their choice (Table 2). Of the 120 participants, 45 (37.5%) chose a young female counselor. Stratified by sex, the results reveal that a low proportion of male participants chose a female counselor aged between 35 and 50 years and a low proportion of female participants chose a male counselor. A chi-square test of association shows some evidence of association between the participant’s sex and the sex of the chosen conversational agent (*P*=.01). This finding revealed that both male and female participants were more likely to select a counselor who was young and of the same sex.

**Table 2:** Preference for counselor among the participants (N=120)^a,b^.

Counselor chosen (age [years]; sex)	Participant sex, n (%)			Total, n (%)
	Male	Female		
18-25; male	16 (13.3)	10 (8.3)	26 (21.7)	
18-25; female	18 (15)	27 (22.5)	45 (37.5)	
35-45; male	18 (15)	6 (5)	24 (20)	
35-50; female	9 (7.5)	16 (13.3)	25 (20.8)	

^a^χ^2^_3_=11.1.

^b^*P*=.01.

## Discussion

### Principal Findings

Acceptance of an HIV self-test using the Nolwazi_bot was assessed in 120 participants. This entailed an assessment of participants acceptance of performing HIVST guided by a chatbot, participants’ ability to interact with the chatbot and follow the instructions of testing, and the chatbots’ ability to guide the participants to conduct HIVST and interpret their results.

This study is one of the first investigations of an mHealth chatbot in South Africa to self-report HIVST results as an outcome outside of a clinical setting. The findings from this study have established that participants showed high acceptability of the chatbot, while also identifying challenges that can be targeted for improvement. The results suggest that some strengths of the chatbot are that it removed time constraints (which is common with a human counselor) and it was empathetic, polite, and educational. Weaknesses of the study include that the almost instantaneous responses of the chatbot were a reminder that it was not human; however, this speed was also noted as an advantage by some participants as it saved time, and 77.5% (93/120) of the participants reported that they felt as if they were talking to a real person. Moreover, the fact that the chatbot was not human was mentioned as an advantage, as the process was viewed as nonjudgmental.

### Strengths of the HIVST Chatbot

Regarding the strengths of the chatbot, an advantage that was mentioned by participants was that they did not feel pressured for time while interacting with the agent, as it allowed testers to carefully answer in their own time and comfort, given that they did not have to worry about wasting another person’s time, especially that of a busy health care worker. In South Africa, a contributing barrier to HIV testing may be rushed HIV counseling services, owing to high patient loads and inadequate facilities [43]. The chatbot may offer an acceptable alternative, which may encourage individuals to conduct HIV testing, who may otherwise not have tested owing to rushed interactions with a health care worker.

Further strengths of the chatbot were illustrated by some participants (15/120, 12.5%) indicating that an advantage of the chatbot was its functionality. These participants highlighted that the chatbot asked the right questions and the counseling was conducted in an empathetic and polite manner and educated them about things they did not know, such as acute HIV infection, PrEP, and information about viral suppression for individuals who are HIV-positive. This finding is of particular importance when considering that 68.3% (82/120) of the participants who tested negative wanted to know more about PrEP.

Another strength that was mentioned was the fast pace of the chatbot agent, as several participants (12/120, 10%) noted that the process can take a mere 30 minutes in comparison with what could take a full day at a PHC clinic. Moreover, another strength of the chatbot that was highlighted by the participants was the perceived safety of the interaction, given that a chatbot would not make any judgments based on their sexual activity. Therefore, chatbots could offer a suitable alternative solution to PHC testing, given that waiting times and issues of privacy have been reported to be barriers to HIV testing in men, who are known to be less-frequent users of public health facilities [44].

Perhaps related to privacy and judgment regarding sexual activity, of the 4 options that participants had for a counselor (man aged 18-25 years, woman aged 18-25 years, man aged 35-50 years, and woman aged 35-50 years), most participants (45/120, 37.5%) selected the woman aged between 18 and 25 years. Interestingly, 40.8% (49/120) of the sample selected an older counselor in comparison with the 59.2% (71/120) of participants who selected a young HIV counselor. Given that 97.5% (117/120) of the participants were aged <35 years, it was hypothesized that most participants would prefer a young counselor. Some participants reported that a reason for the selection of an older counselor was the perception that with increased age, there is increased wisdom, which in turn would benefit the recipient of counseling. Given the limited sample size, no concrete conclusions can be drawn beyond these simple observations. Of significance for this study is that most participants (93/120, 77.5%) reported that their HIV testing experience with a chatbot was much better than that with a human counselor. Considering the abovementioned human resource limitations in the South African health care system, using mHealth tools in HIVST could contribute to alleviating the current demands on the health care system. Although further studies and development are still required to understand the potential uses, legal implications, and impact of conversational agents in health care, the data suggest that with improvement, chatbots may be able to provide public health screening not only for chronic infectious diseases (such as HIV) but also for noncommunicable diseases (such as diabetes and hypertension) in low-resource settings.

### Weaknesses of the HIVST Chatbot

If one looks at the weaknesses of the chatbot, a disadvantage that was illustrated in the results is that the chatbot replied very fast, and therefore, the responses were said to be not human-like. This was mentioned as a disadvantage by 12.5% (15/120) of the participants. The same finding was noted in a similar study using the less-advanced Nolwazi_bot mHealth counseling agent [30]. With further programming, this perceived disadvantage can be overcome. For example, the chat agent responses could be made to be more human-like by delaying how quickly a response is sent and simulating slow (human-like) typing speeds. However, it may be worthwhile to give participants the option at the beginning of the conversation to indicate whether they are in a rush, as several participants (12/120, 10%) indicated that an advantage of the HIVST process with the chatbot was that it was a quicker process than that ordinarily done with a human counselor, and a contributing factor to this speed would be the almost immediate response time from the chatbot. Furthermore, the perceived disadvantage of rapid responses was only noted by a minority of the participants, and it should be noted together with the finding that 77.5% (93/120) of the participants reported that they felt as if they were talking to a real person.

Despite the apparent advantages of the HIVST chatbot in comparison with a human counselor, it is necessary to be cognizant that artificial intelligence is limited in its ability to weigh competing personal values and to be conscientious [45]. Parviainen and Rantala [46] argue that the intelligence of chatbots cannot assess emergency health situations and may cause harm owing to the lack of knowledge of personal factors associated with specific patients. This is particularly pertinent when one considers the range of emotions that one may be experiencing upon receiving an HIV-positive result. Nevertheless, when one considers that an individual may not go to a clinic for an HIV test owing to fear related to stigma, long waiting cues, or other concerns, it would be more beneficial for someone to perform a home HIV test with an mHealth conversational chatbot than to not test and not be aware of their (potential) HIV-infected status.

### Limitations and Strengths

The study has several limitations. First, the generalizability of the findings is limited because convenience sampling was used, and participants were recruited from 1 subdistrict of KwaZulu-Natal. It would be beneficial for similar studies conducted in the future to have a large sampling frame to improve the generalizability of the findings. Second, another limitation to generalizability is that only 1 HIVST kit was used; therefore, these results cannot be generalized across all HIVST kits. Future studies would benefit from using several HIVST kits. Third, most participants (103/120, 85.8%) were aged <30 years, which may have increased the acceptability and ease of navigation of the chatbot, as young individuals are likely to have more technological skills than older age groups. Fourth, the chatbot was only tested on a Samsung phone and Telegram. This may not reflect the usability of the chatbot on other phones and platforms such as WhatsApp, which has a higher download rate on Google Play in South Africa than Telegram. Fifth, recent studies have developed validated data collection methods to determine the usability of mHealth tools [47]. The last limitation of the study is that the authors used an adapted, unvalidated scale to assess usability. Unfortunately, resources were not available to perform cognitive interview or other validation procedures before its use. The results from the study-specific questions of this study may not be replicable in similar settings, as (to the best of our knowledge) there are no validated data collection tools to assess the acceptability and feasibility of mHealth for HIVST.

The study had some strengths. First, 92.5% (111/120) of the participants have completed ≥2 HIV tests with a human counselor, which can be argued to contribute to the face validity of the abovementioned results, as the participants can be said to be well placed to compare the chatbot with a human counselor and therefore determine the strengths and weaknesses of the chatbot in relation to a human counselor. Second, the sample included a good representation of participants with various levels of education, including tertiary level, secondary or high school level, primary school level, and even no education. Third, the sample had a good representation of both men and women with the distribution being approximately balanced.

### Conclusions

Although we wait to see the full benefits of mHealth, technological interventions including conversational agents or chatbots provide us with a good opportunity to improve HIVST, by addressing some of the barriers faced by both facilities and patients. The study provides insights into the potential of digital technological interventions to support health to improve HIVST, by addressing the barriers associated with clinic-based HIV testing.

## Appendix

Multimedia Appendix 1 Posttest structured survey.

## Abbreviations

HIVST: HIV self-testing
HTC: HIV testing and counseling
HTS: HIV testing service
mHealth: mobile health
NLU: natural language understanding
PHC: primary health care
PrEP: pre-exposure prophylaxis

## Data Availability

The data sets generated and analyzed during this study are available from the corresponding author upon reasonable request.
