# Supplementary material for: The Feasibility and Acceptability of an mHealth Conversational Agent Designed to Support HIV Self-testing in South Africa: Cross-sectional Study
Source: J Med Internet Res. 2022 Dec 12;24(12):e39816. doi: 10.2196/39816 (PMC9793294; doi:10.2196/39816)
Supplement: Multimedia Appendix 1 [file jmir_v24i12e39816_app1.pdf]

Exit Survey

1. Please enter the date on which this interview is taking place. ___________________
2. Interviewer Name_________________
3. Record participants gender. Male / Female.
4. What is your date of birth? __________________
5. What is your highest level of completed education?
    a. Primary school [1]
    b. Secondary/high school [2]
    c. Tertiary institution [3]
    d. None [4]
6. Does the household have any of the following items in good working order? Select all that apply.
    a. Cellphone
    b. Primus Stove
    c. Electric hotplate
    d. Electric stove with oven
    e. Gas stove
    f. Fridge of freezer
    g. Electric kettle
    h. Television
    i. DVD player
    j. Radio
    k. Sewing machine
    l. Car or bakkie
    m. Motorcycle
    n. Bicycle
    o. Bed
    p. Table and chairs
    q. Lounge suite
    r. Kitchen sink
    s. Wheelbarrow
    t. Hoe, spade or garden fork
    u. Cattle
    v. Other livestock
    w. Computer with internet
    x. Satellite TV
7. How many times have you ever tested for HIV?
    a. 1
    b. 2-5 times
    c. 5-10 times
    d. 10+ times

8. How would you compare this experience with Nolwazi to a session with a human counsellor?
    a. Much worse
    b. Slightly worse
    c. About the same
    d. Slightly better
    e. Much better
9. Did it feel like a real person was replying? Why/why not?
10. Would you consider doing a real test with Nolwazi? Why/why not?
11. Would you prefer a male or female conversational agent and why?
12. Could you list any advantages you see to having a chatbot offer you counselling as compared to a human counsellor?
13. Could you list any disadvantages you see to having a chatbot offer you counselling as compared to a human counsellor?
14. On a scale from 1 to 10 (where one is poor and 10 is excellent) how would you rate the counselling experience?
15. What was your biggest concern going into the study?
16. What did your view change now that you have finished the study?
